# Real-World Performance of a New Online Eye Symptom Triage Tool (Eye+Dot) in an Emergency Eye Clinic: Mixed Methods Evaluation Study

**DOI:** 10.2196/81343

**Published:** 2026-04-14

**Authors:** Louise Allen, Jen O Lim, Fengyi Liu, Nimesha Alex

**Affiliations:** 1 Department of Ophthalmology, Cambridge University Hospitals NHS Foundation Trust, Cambridge Biomedical Research Campus, Hills Road, Cambridge, CB2 0QQ, United Kingdom, 44 1223 216700; 2 University of Cambridge, Cambridge, United Kingdom; 3 School of Clinical Medicine, University of Cambridge, Cambridge, United Kingdom

**Keywords:** triage, triage methods, mobile apps, computer-assisted decision-making, decision tree, emergency service, eye diseases, optometry, mobile health, digital health

## Abstract

**Background:**

Previous studies indicate that 37% to 92% of patients presenting to hospital emergency eye clinics (EECs) could be seen in commissioned community optometrist enhanced service schemes (ESSs), reducing pressure on hospital services and moving eye care into the community. Digital triage tools may have the potential to support effective triage and use of ESSs.

**Objective:**

This study sought to evaluate the effectiveness of a recently developed online symptom triage tool in real-world EEC practice and assess its usability and acceptance by patients.

**Methods:**

This was a prospective, real-world study comparing automated dispositions suggested by the eye+dot online triage tool with nursing triage, using ophthalmologist dispositions and subsequent clinical findings as the reference standard. Patients aged 13 years and older were sent an SMS text message invitation to use eye+dot prior to their scheduled EEC appointment. Age, time required to complete the symptom questionnaire, and acceptability (using an in-application visual Likert scale) were also recorded. The accuracy of the different triage methods at assigning patients to eye assessments within 24 hours, 48 to 72 hours, or a week based on symptom urgency was compared. Eye+dot’s accuracy in identifying patient suitability for ESSs was analyzed.

**Results:**

A total of 282 eligible patients with a mean age of 53.14 (SD 19.8; range 13-92) years were included. The mean eye+dot test duration was 5.6 (SD 2.4) minutes, with 80.4% (168/209) of patients rating the test as good or excellent. For high-acuity symptomatology (defined via retrospective ophthalmologist recommendation for assessment within 24 hours), sensitivity was similar between eye+dot and nurse triage (58/76, 76.3%; 95% CI 65.2%-85.3% and 65/76, 85.5%; 95% CI 75.6%-92.5%, respectively; *P*=.19). However, eye+dot specificity was significantly higher (132/206, 64.1%; 95% CI 57.1%-70.6% vs 47/206, 22.8%; 95% CI 17.3%-29.2%; *P*<.001). Of 224 urgent nursing dispositions, 203 (90.6%) patients were downrated (considered suitable for nonurgent assessment) by ophthalmologist assignment, and 93 (41.5%) were downrated by eye+dot. In total, 90.8% (256/282) of the patients were considered suitable for ESS assessment through eye+dot triage. A total of 56.6% (145/256) of these patients subsequently received only medical advice without specialist investigations or management at their EEC visit, suggesting suitability for community eye care.

**Conclusions:**

Eye+dot–automated triage has similar sensitivity but superior specificity to nursing triage for identifying high-acuity symptomatology. This small study suggests its potential to improve patient scheduling in EECs throughout the working week and improve the use of community services. A larger study is planned to establish the utility of the triage tool and develop an implementation model to scale up and spread the technology.

## Introduction

The demand for urgent eye care in the United Kingdom continues to rise, adding to capacity pressures in hospital emergency services. New-patient eye care attendance is estimated at 20 to 30 per 1000 person-years, and ophthalmic emergencies make up 1.4% to 6% of emergency department (ED) attendances [1]. Primary care triage staff may tend to be risk averse in referring patients to hospital services, and studies of emergency eye clinic (EEC) attendance demonstrate that 37% to 92% of referred patients could have been seen in community enhanced service schemes (ESSs), including minor eye condition services or community urgent eye services, or scheduled outpatient eye clinics [2-5]. Redirecting suitable patients from hospital to community care is a key component of the Royal College of Ophthalmologists emergency eye care commissioning guidelines and the UK National Health Service (NHS) 10-year plan [6-8].

Triage support charts are used in many EECs to rate symptom acuity and guide urgency disposition for less experienced triage staff. Given the pressures on emergency services, interest in digital support tools is increasing. Active public-facing UK websites that signpost patients to eye care services include EuroEyes, NHS inform, and the NHS 111 Wales Eye Problems Symptom Checker tool. New clinician-facing triage support digital platforms, such as DemDx and ASSORT (Artificial Intelligence–Based Symptom Stratification in Ophthalmology for Rapid Triage), use machine learning and large language models to support triage decisions. Eye+dot is a new online triage tool that effectively functions as a digital symptom acuity chart using Royal College of Ophthalmologists triage urgency recommendations for use by patients at the direction of the triage provider. Patients complete an online multiple-choice questionnaire, which then provides the triaging service or clinician with a symptom report, suggestions as to the urgency with which the patient should be seen, and which eye care service provider would be suitable.

The objectives of this study were to evaluate the effectiveness of the eye+dot triage tool in real-world EEC practice and assess its usability and acceptance by patients.

## Methods

### Eye+Dot Triage Tool

Eye+dot has been designed for evaluation of recent-onset (within 1 week) ophthalmic symptoms and is suitable for patients aged 13 years and over. To undergo the test, patients use a web link contained in an invitation sent via email or SMS text message. Where eye+dot has been integrated, the invitation can be sent using an order from the organization’s electronic patient record (EPR). The test consists of a branching logic questionnaire where each response determines the next question or question sequence. Each patient is asked a maximum of approximately 25 questions covering symptomatology, ophthalmic history, and medication use. On completion, the symptom report and suggested disposition can be viewed by the triage provider using a link either sent via email or available in the EPR. The suggested disposition includes both urgency (requiring assessment within the same day, 24 hours, 48-72 hours, or a week) and the most suitable eye care service (ED or EEC, hospital or community urgent eye service, private optometry, or pharmacy). The patient does not see the report or suggested triage disposition. However, if their responses warrant it (eg, a history of recent sight-threatening injury, recent sudden loss of vision, or symptoms of stroke), the patient is fast-tracked to the end of the questionnaire and receives a link to online NHS advice, including first aid and need for immediate ED attendance. Other than age, eye+dot does not collect any personal data, and the results must be linked to the patient by the triage provider (or their EPR) using the unique order reference provided when the invitation is sent.

The eye+dot web application (Cambridge Medical Innovation Ltd) has been developed and tested over several sequential proof-of-concept and validation studies based on simulated and real-life scenarios in 3 hospital trusts of the Cambridgeshire and Peterborough Integrated Care Service and, most recently, in minor eye condition service optometry practices 9. The initial process involved the development of multiple-choice questions, with the question set, branching logic structure and triage disposition algorithm being iteratively improved over a series of patient feedback studies since the application was first developed in 2021.

**Figure 1.**
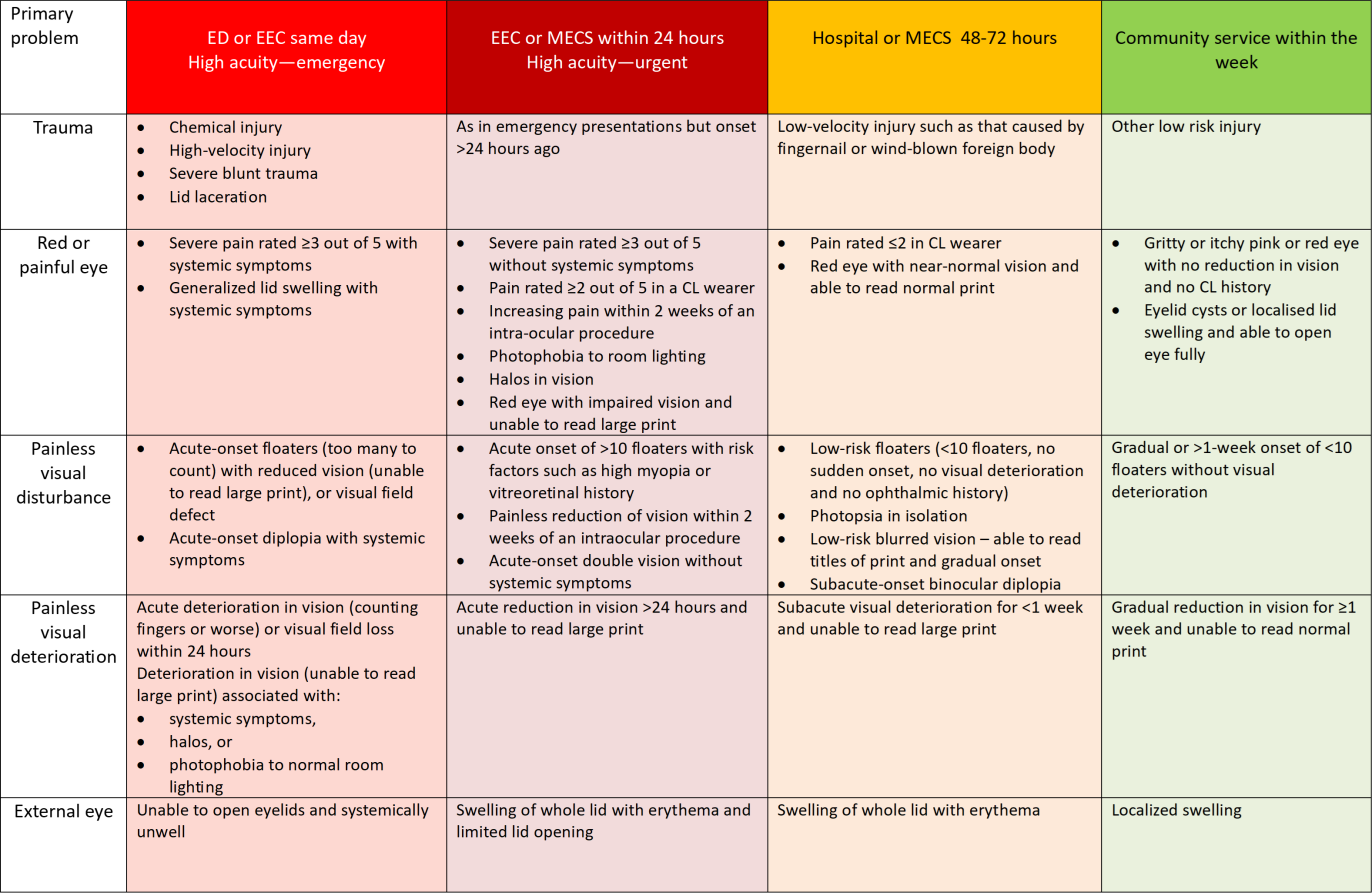
Symptom triage support chart (adapted from the Royal College of Ophthalmologists emergency eye care guidelines) [7]. CL: contact lens; ED: emergency department; EEC: emergency eye clinic; MECS: minor eye condition service

The triage tool uses only self-reported symptoms and history, and the disposition algorithm is based on the scoring system used by accepted published chart-based guides (Figure 1 [7]). Integration with an EPR (Epic) enables clinicians to order and retrieve results directly through the patient record. The application is currently in clinical use in the NHS.

### Study Design

This was a prospective, single-center, real-world evaluation study that compared eye+dot triage dispositions with those generated by the standard nursing triage process. Independent triage decisions made by ophthalmologists based on their interpretation of the eye+dot symptom report were used as the standard of reference. Subsequent tests, diagnosis, and management at the scheduled EEC appointment were documented and reviewed.

The EEC at our NHS trust does not offer walk-in appointments, although patients already under the care of the trust’s ophthalmology department can self-refer to this EEC. More commonly, patients are referred from primary care services via email or phone call. The specialist nurse triage team phones the patients, assesses their history, and triages them to either a scheduled EEC session or out-of-hours assessment by the on-call team. Currently, no patients are referred to local ESS optometry practices by nursing triage.

**Figure 2.**
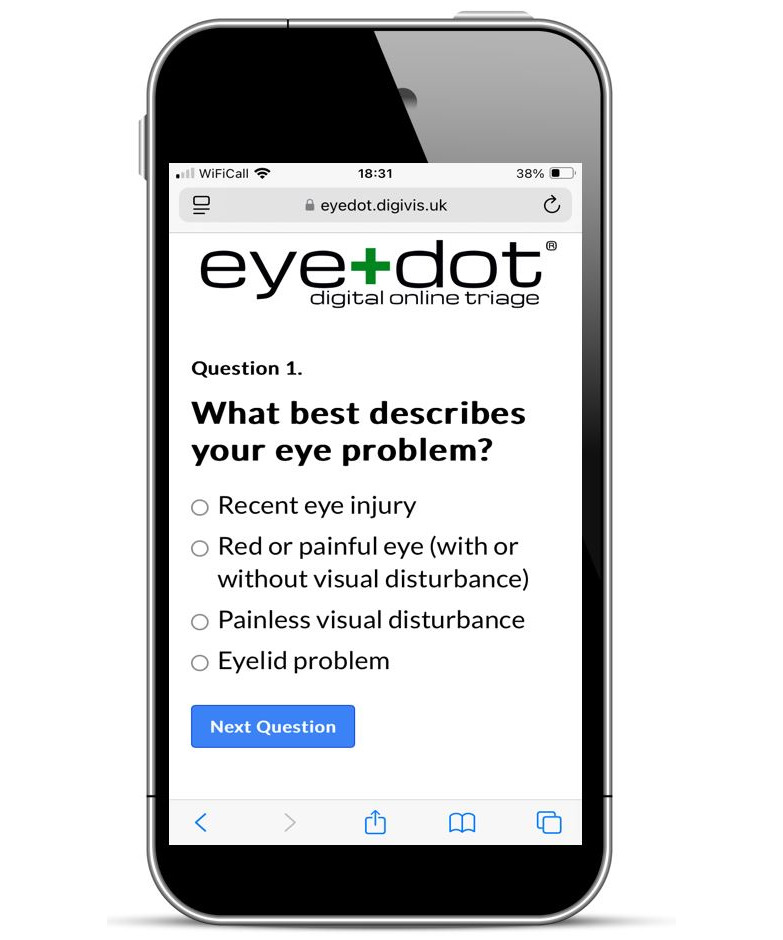
Initial eye+dot symptom filter question as seen by the patient.

**Figure 3.**
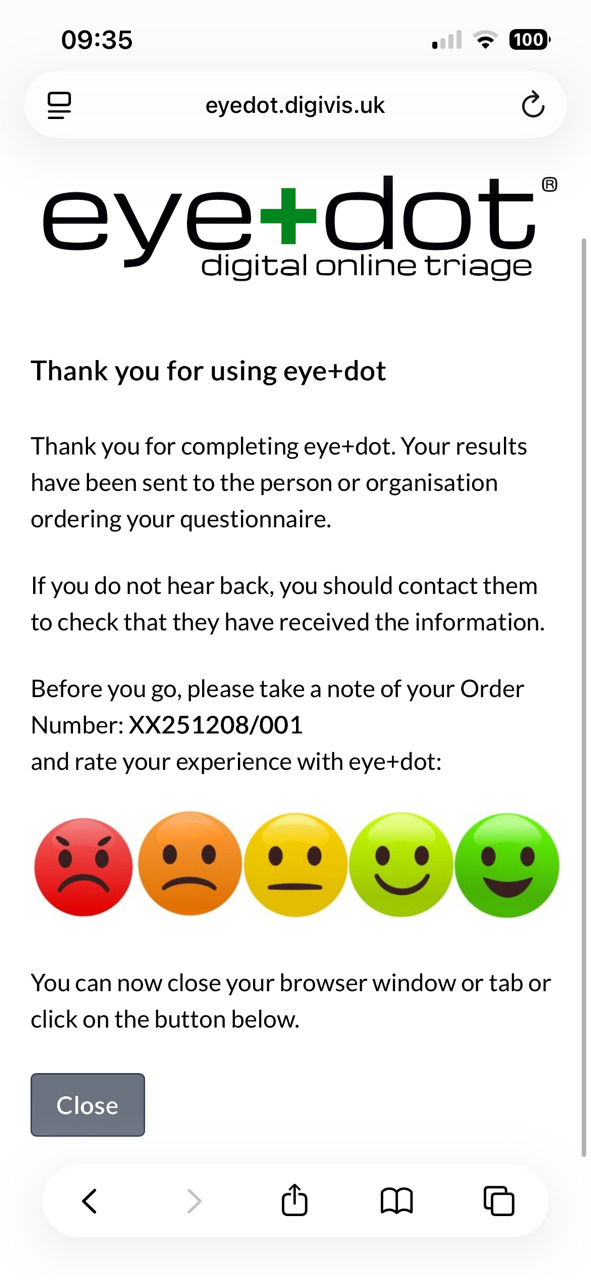
Visual Likert scale presented to patients after completion of the eye+dot questionnaire.

Over a 3-month period, patients aged 13 years and older with a documented mobile phone number who had scheduled appointments in EEC clinics were sent an eye+dot invitation via SMS text message prior to attendance. Patients accessing the eye+dot questionnaire were also asked to accept a General Data Protection Regulation–compliant data use notice. Data collected from the eye+dot system included the selected primary concern on the initial filter question (Figure 2), the compiled symptom report, suggested disposition, test duration, and visual Likert-scale score of acceptability (optional element of the eye+dot questionnaire; Figure 3).

At the end of the 3-month period, records of patients who completed eye+dot triage were reviewed. Tertiary referrals, inpatient referrals, and patients referred with known diagnoses were excluded from analysis. For the remaining patients, the urgency assigned at nursing triage was documented and classified; this was defined as urgent for patients assigned for assessment on the same day (A) or within 24 hours (B), semiurgent for patients assigned for assessment within 48 to 72 hours (C), and lower urgency for patients assigned for assessment within the week (D). For the purposes of our analysis, patients were considered suitable for community care if no imaging or other diagnostic tests, prescription medication, or surgical procedures were required at the time of the EEC appointment.

To provide a reference standard, 2 ophthalmologists (a consultant and year 7 resident physician) masked to the other triage suggestions or decisions reviewed the eye+dot ophthalmic symptom report and graded the urgency using the A-to-D urgency grading criteria described above. In cases of disagreements between ophthalmologist reviewers, the higher-urgency disposition was used as the reference standard. Where a categorization of urgent by both ophthalmologists was not matched by the eye+dot and/or nursing dispositions, patient case notes were reviewed to determine whether the undertriage by eye+dot or nurses might have affected clinical outcomes.

### Ethical Considerations

This service evaluation study was approved following ethical consideration by the Quality and Safety Committee at Cambridge University Hospitals NHS Foundation Trust and was conducted in accordance with the tenets of the Declaration of Helsinki. As an anonymized service evaluation study not involving randomization or deviation from the current standard of clinical care, this study was exempt from the requirement for an explicit informed consent process and national ethics committee approval (PRN13310) [10]. Patients could choose not to participate without any impact on their EEC appointment or care. The eye+dot software application replicates a chart-based symptom scoring chart and, thus, does not meet the criteria for classification as a medical device under the UK Medical Devices Regulations based on Medicines and Healthcare Products Regulatory Agency guidance [11,12].

### Statistical Analysis

Patients’ ages, test durations, and Likert-scale scores were analyzed using descriptive statistics. The frequency distributions of triage dispositions were compared between raters. Interrater agreement was analyzed using the percentage of agreement and quadratically weighted Cohen κ as the magnitude of disagreement in triage urgency categories is likely nonlinearly related to the resulting clinical risk for most patients. A priori interpretation of κ values was as follows: values of 0 or lower indicated no agreement, values of 0.01 to 0.20 indicated none to slight agreement, values of 0.21 to 0.40 indicated fair agreement, values of 0.41 to 0.60 indicated moderate agreement, values of 0.61 to 0.80 indicated substantial agreement, and values of 0.81 to 1.00 indicated almost perfect agreement [13]. The accuracy of eye+dot and nurse triage dispositions at identifying cases triaged as urgent (criterion A or B) by at least one ophthalmologist reviewer was calculated, and the difference in sensitivity and specificity for urgent cases was evaluated using the McNemar test with continuity correction. The number and proportion of lower-urgency patients (criterion C or D) correctly identified by eye+dot as being suitable for community ESSs was also assessed. Feedback from patients provided through a visual Likert scale (Figure 3) was converted into numerical values from 1 to 5 for analysis.

## Results

### Overview

**Figure 4.**
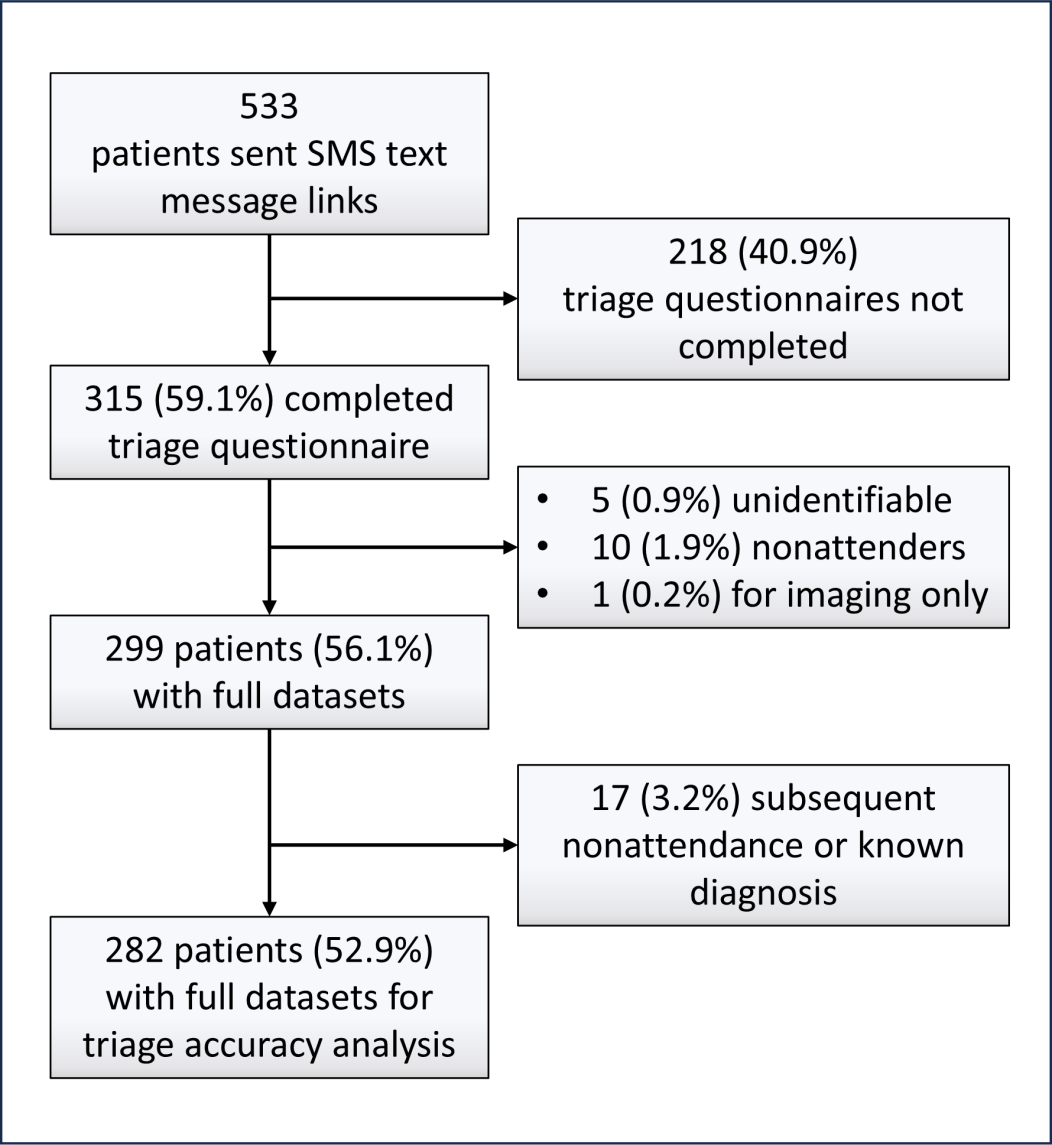
Study flowchart.

Of 533 patients sent eye+dot questionnaire links, 319 (59.8%) started the questionnaire, and 315 (98.7%) of these patients completed it. In total, 33 (6.2%) patients were excluded from the subsequent analysis: of whom 17 (51.5%) already had a diagnosis at the time their appointment was scheduled, 5 (15.2%) had incomplete documentation, 10 (30.3%) did not attend, and 1 (3.0%) attended the EEC for imaging only. Therefore, full datasets from 282 (52.9% of total 533) eligible patients were available for analysis (Figure 4). Among these patients, red or painful eye and painless visual disturbance were the most common presenting concerns (Table 1).

**Table 1.** Patients included for analysis by presenting concern based on response to the first question of the eye+dot questionnaire (Figure 2; N=282).

Presenting concern	Patients, n (%)
Red or painful eye	141 (50.0)
Painless visual disturbance	116 (41.1)
Eye injury	18 (6.4)
Eyelid problem	7 (2.5)

### Age of Patients and Eye+Dot Acceptability

The mean ages of the patients completing eye+dot triage compared with those who chose not to use it were similar: 53.14 (SD 19.8; range 13-92) years as opposed to 53.2 (SD 19.8; range 13-94) years, respectively.

A total of 80.4% (168/209) of the patients who gave voluntary usability feedback rated the application with a 4 or 5 (with 5 representing the most positive rating) on the visual Likert scale. The mean Likert-scale score was 4.21 (SD 0.82; range 1-5). Mean test duration was 5.6 minutes (SD 2.4 minutes; range 9 seconds-20.7 minutes).

### Interrater Agreement on Urgency

**Figure 5.**
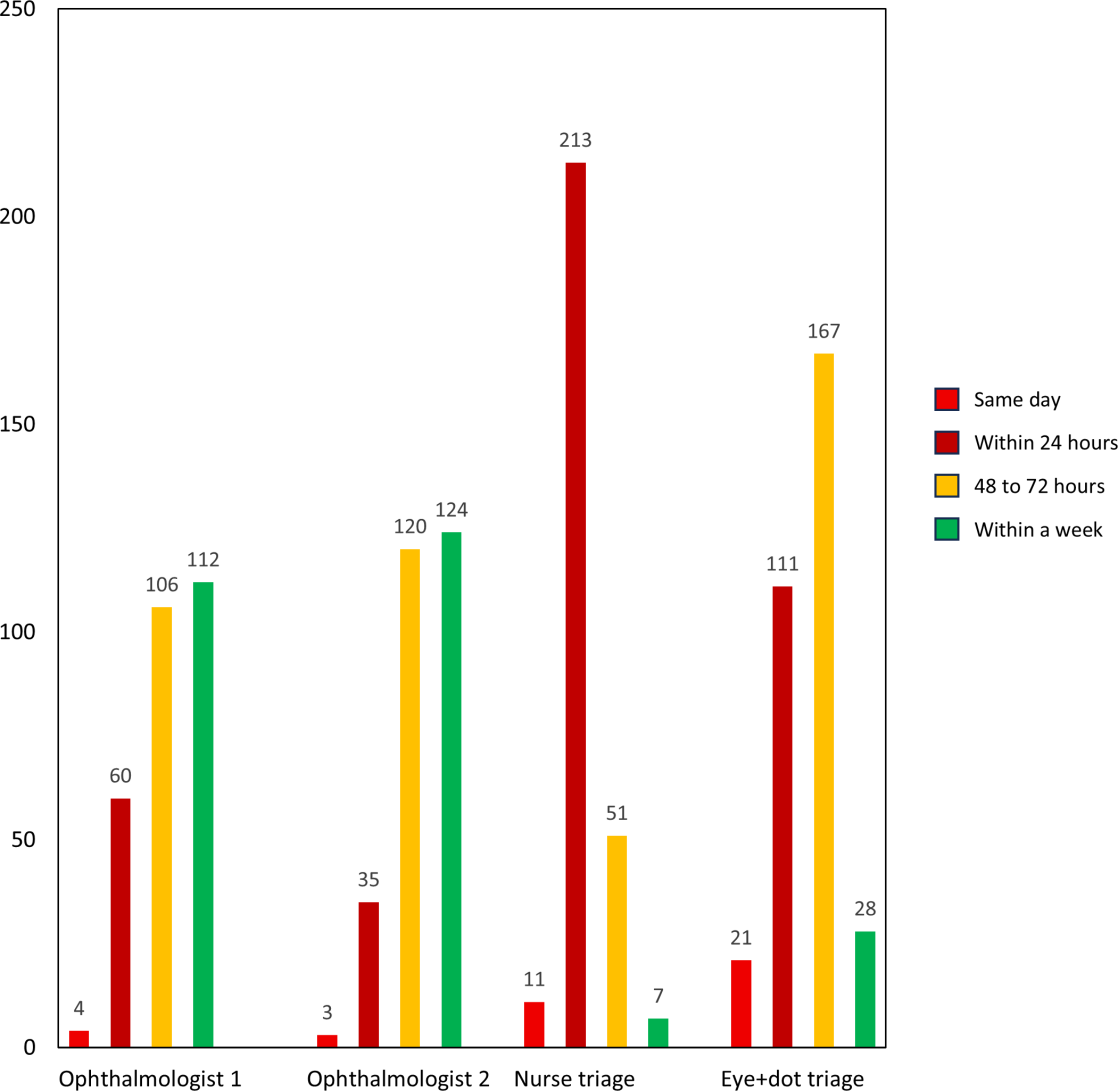
Frequency distribution of rater urgency gradings

The frequency distribution of urgency assignments for each rater (grades A-D) is shown in Figure 5.

There was fair agreement (κ=0.22) between the 2 ophthalmologists and between ophthalmologist 1 and eye+dot (κ=0.38), but otherwise, there was only slight or almost no agreement (κ=0.01-0.2) between other raters (Table 2). Eye+dot overestimated the urgency compared with the combined ophthalmologist grading in 36.5% (103/282) of the patients and underestimated it in 1.4% (4/282); nursing triage overestimated the urgency in 59.6% (168/282) of the patients and underestimated it in 3.2% (9/282).

**Table 2.** Interrater agreements for urgency gradings A to D and percentage of agreement for urgent grades (A or B).

Raters	Cohen κ (95% CI)	Agreement with urgent grading (%)
Ophthalmologist 1 vs ophthalmologist 2	0.22 (0.10 to 0.34)	78.4
Ophthalmologist 1 vs nurse triage	0.06 (0.01 to 0.11)	37.6
Ophthalmologist 1 vs eye+dot	0.38 (0.30 to 0.47)	71.3
Ophthalmologist 2 vs nurse triage	0.01 (–0.04 to 0.06)	29.1
Ophthalmologist 2 vs eye+dot	0.05 (–0.04 to 0.13)	56.5
Eye+dot vs nurse triage	0.03 (–0.06 to 0.11)	47.9

### Accuracy in Identifying Urgent Cases

Of the total 282 patients, 224 (79.4%) and 132 (46.8%) were assigned an urgent triage disposition (A or B) through nursing and eye+dot symptom triage, respectively, compared with 76 (27%) who were assigned an urgent triage disposition by at least one ophthalmologist. There was no significant difference (*P*=.19) in the sensitivity of nursing and eye+dot triage in identifying urgent cases as defined by ophthalmologist review, but there was a significant difference (*P*<.001) in specificity (Table 3). The percentage of agreement of nursing and eye+dot triage with cases graded as urgent by one or both ophthalmologists was 39.7% and 67.4%, respectively.

**Table 3.** Sensitivity and specificity of nurse and eye+dot triage for identifying urgent cases (compared with the reference-standard grades of A or B assigned by one or both ophthalmologist reviewers). *P* values were calculated using the McNemar test (N=282).

	Nurse triage vs reference-standard urgent grades (%; 95% CI)	Eye+dot triage vs reference-standard urgent grades (%; 95% CI)	*P* value
Sensitivity	85.5 (75.6-92.5)	76.3 (65.2-85.3)	.19
Specificity	22.8 (17.3-29.2)	64.1 (57.1-70.6)	<.001

### Clinical Presentations and Outcomes of Urgent Cases

Further analysis was undertaken in the subset of 7.4% (21/282) of patients whom both ophthalmologists classified as urgent. In total, 19% (4/21) of these patients had been undertriaged by nursing or eye+dot triage. A total of 4.8% (1/21) of the patients’ triage decisions were uprated by the ophthalmologists from both the nurse and eye+dot dispositions of C (assessment within 48 hours). This patient had added “I have a foreign object in my upper lid which is now protruding over my eyelashes and it’s now blocking my vision in my right eye” in the free-text entry, which was visible to the ophthalmologists on the eye+dot report. However, upon review, this patient’s injury had been sustained 1 year before, and he had a retained glass foreign body in the eyelid since that time; this history had been elicited during nursing triage.

Nursing triage was uprated from C to B in a further 14.3% (3/21) of the patients: one had a resolving corneal abrasion, the second had suspected migrainous aura with associated temporal tenderness, and the third had ophthalmic shingles with corneal involvement.

Of the 224 urgent nursing triage dispositions, 203 (90.6%) patients were downrated by ophthalmologist review, and 93 (41.5%) were downrated by eye+dot.

### Service Disposition

In total, 46.5% (131/282) of the patients who attended the EEC did not require hospital diagnostic or medical management and, thus, were considered to have been suitable for community care through ESSs.

Eye+dot suggested suitability for ESSs in 90.8% (256/282) of the patients. A total of 56.6% (145/256) of these patients required assessment and advice only during their subsequent EEC visit and may have been suitable for community optometrist care. The other 43.4% (111/256) of the patients received diagnostic imaging or medical management in the EEC and may potentially have needed subsequent referral to hospital eye services (HESs) had they been seen in ESSs initially.

## Discussion

### Principal Findings

Triage systems should ideally have both high sensitivity and high specificity to ensure timely access to care and efficient use of resources. Triage accuracy particularly depends on factors such as clinical experience, evaluation time, and the use of support tools or frameworks [14,15].

A recent large retrospective study at Moorfields Eye Hospital ED reported nurse triage sensitivity of 96.4% and specificity of 25.1% for identifying emergency cases among patients attending the EEC [16].

This real-world validation study found similar results for nurse-led triage in relation to patients assigned an urgent grading, but the eye+dot triage support tool, although it had similar sensitivity (*P*=.19), showed significantly higher specificity for emergency cases than nursing triage (*P*<.001).

These results suggest that use of eye+dot could have safely downrated the triage category in 41.5% (93/224) of patients assigned as urgent via nurse triage, potentially enabling redistribution of patient load throughout the working week and redirecting 51.4% (145/282) of patients from a planned EEC attendance to a community ESS appointment without the risk of a subsequent rebound referral back to the HES.

### Comparison of Interrater Agreement to That in Prior Studies

It is notable that the interrater agreement in this study was poor between most triage modes (optometrists, eye+dot, and ophthalmologist reviewers), and was only fair between the 2 ophthalmologists (κ=0.22, 95% CI 0.10-0.34) and between eye+dot and the consultant ophthalmologist (κ=0.38, 95% CI 0.30-0.47). This stands in contrast to interrater agreements reported in other studies using digital triage tools. A real-world validation study at Scheie Eye Institute, University of Pennsylvania, reported excellent interrater agreement between their online symptom-based triage tool and reference standard (κ=0.91). However, this may be because prioritization categories were in wider, more discrete urgency bands of “emergency/urgent (same day),” “semi-urgent 1-4 weeks,” and “nonurgent/elective” compared with the relatively narrow urgency categories used in this study [17]. Studies using large language models have also claimed relatively high interrater agreement (κ=0.773-0.751) for their models using simulated clinical scenarios, but this may not reflect the complex and sometimes inconsistent symptom reporting often encountered in the real world [18-20]. A recent pilot study of the GPT-4–powered ASSORT triage tool in 51 patients presenting in an EEC with concerns requiring prompt evaluation showed moderate interrater agreement between ASSORT and the triaging ophthalmologist (κ=0.54) with overestimation of severity [21]. In that cohort, 56.8% of patients were considered urgent by the ophthalmologist compared with the 27% (76/282) in our study population of nurse-triaged scheduled attenders, suggesting that the populations may not be comparable.

### Utility of the Triage Tool as an Aid for Redirecting Patients to Community Services

Using the eye+dot triage tool, all patients with newly reported recent-onset symptoms would have had a professional eye assessment within the week, but the urgent workload would have been more evenly distributed, with most patients (256/282, 90.8%) potentially being assessed in the community ESSs first.

Of these, 43.4% (111/256) may have required diagnostic imaging or medical management, necessitating a subsequent rebound referral to HESs, although this is likely to be an overestimation as many ESS providers now have diagnostic imaging and prescribing optometrists available. A 2016 study reported that only 18.9% of 2123 patients attending ESSs in a South London area required a subsequent referral to HESs [22].

This potential benefit of redirecting patients to community ESSs before hospital assessment cannot be fully realized by some other triage tools which require the input of nurse-elicited symptoms and signs. Examples include DemDx, the Alphabetical Triage Score for Ophthalmology, and the Rome Eye Scoring System for Urgency and Emergency [16,23,24].

### Usability Feedback and Potential Workflow Issues

A total of 59.8% (319/533) of the patients who were sent invitations clicked on the eye+dot link in the SMS text message, which we consider to represent a reasonable openness to engaging with the new technology, given that they had received no prior notification to expect this. Providing advance information regarding eye+dot at the time of initial contact might have increased uptake. The eye+dot application was generally well accepted and was rated within the top 2 out of 5 possible Likert scale options by 80.4% (168/209) of the patients in this study who responded to the request for feedback at the end of the questionnaire. All had already been scheduled for an EEC appointment, which may have influenced their acceptance, and it is possible that reported usability of the tool may decrease if the information collected is used to redirect patients to community services. A related potential issue, which requires evaluation in practice, is whether patients who had already been referred to the EEC by their primary care team would be resistant to being redirected to community eye care providers based on this triage process. Both ESSs and EECs are free-to-access NHS services, but patient resistance to triage advice based on financial cost may be a necessary factor to consider in other countries or contexts. NHS England is developing single point of access call centers for patients with recent-onset symptoms to be triaged and directed to a suitable service. This may be the ideal point to implement specialist digital triage tools such as eye+dot to prevent the patient from being bounced between health care providers.

There was no difference in age between those who responded to the questionnaire and those who did not; however, it is possible that older patients without mobile phone access may have been underrepresented. Systematic reviews of patient experience of primary care online triage describe the importance of issues of digital and literacy inequality, sight impairment, concerns regarding privacy, and distrust of online platforms. Some patients find that the convenience and speed of online triage platforms outweigh their transactional and impersonal nature, and for others, it is quite the reverse [25,26]. Access to the technology for the visually impaired may be considered a particular limitation for an eye symptom triage tool, although this is mitigated by the ease of responding to the eye+dot questionnaire over the telephone. None of the patients in this study cohort were identified as visually impaired, possibly because patients with preexisting eye conditions are more likely to have direct lines of access to specialist care and be less likely to use a primary triage tool. For staff, although there may be concerns that greater use of digital triage tools could lead to deskilling or even job losses, given the current staffing shortages and ever-increasing workload, this seems unlikely. Both they and patients may benefit from the advantages and support of an automated questionnaire that can be completed in the patients’ own time and records important red-flag and negative symptomatology that can be documented in the EPR.

### Conclusions

This real-world study demonstrates that eye+dot triage has similar sensitivity to but significantly higher specificity than nursing triage in assessing urgency for patients scheduled for EEC appointments. An implementation study is planned to determine its utility in safely downtriaging and redirecting appropriate cases to community eye care services and the effect this has on ED and EEC service efficiency and to develop a model for the spread of the technology.

## Abbreviations

ASSORT: Artificial Intelligence–Based Symptom Stratification in Ophthalmology for Rapid Triage
ED: emergency department
EEC: emergency eye clinic
EPR: electronic patient record
ESS: enhanced service scheme
HES: hospital eye service
NHS: National Health Service
